# Profiling mRNAs of Two *Cuscuta* Species Reveals Possible Candidate Transcripts Shared by Parasitic Plants

**DOI:** 10.1371/journal.pone.0081389

**Published:** 2013-11-27

**Authors:** Linjian Jiang, Asela J. Wijeratne, Saranga Wijeratne, Martina Fraga, Tea Meulia, Doug Doohan, Zhaohu Li, Feng Qu

**Affiliations:** 1 Department of Horticulture and Crop Science, Ohio Agricultural Research and Development Center, The Ohio State University, Wooster, Ohio, United States of America; 2 Department of Plant Pathology, Ohio Agricultural Research and Development Center, The Ohio State University, Wooster, Ohio, United States of America; 3 Molecular and Cellular Imaging Center, Ohio Agricultural Research and Development Center, The Ohio State University, Wooster, Ohio, United States of America; 4 Wooster High School, Wooster, Ohio, United States of America; 5 State Key Laboratory of Plant Physiology and Biochemistry, College of Agronomy and Biotechnology, China Agricultural University, Beijing, China; Universidad Miguel Hernández de Elche, Spain

## Abstract

Dodders are among the most important parasitic plants that cause serious yield losses in crop plants. In this report, we sought to unveil the genetic basis of dodder parasitism by profiling the trancriptomes of *Cuscuta pentagona* and *C. suaveolens*, two of the most common dodder species using a next-generation RNA sequencing platform. *De novo* assembly of the sequence reads resulted in more than 46,000 isotigs and contigs (collectively referred to as expressed sequence tags or ESTs) for each species, with more than half of them predicted to encode proteins that share significant sequence similarities with known proteins of non-parasitic plants. Comparing our datasets with transcriptomes of 12 other fully sequenced plant species confirmed a close evolutionary relationship between dodder and tomato. Using a rigorous set of filtering parameters, we were able to identify seven pairs of ESTs that appear to be shared exclusively by parasitic plants, thus providing targets for tailored management approaches. In addition, we also discovered ESTs with sequences similarities to known plant viruses, including cryptic viruses, in the dodder sequence assemblies. Together this study represents the first comprehensive transcriptome profiling of parasitic plants in the *Cuscuta* genus, and is expected to contribute to our understanding of the molecular mechanisms of parasitic plant-host plant interactions.

## Introduction

Parasitic plants receive part or all of the nutrients, including photosynthate, nitrogen, minerals, as well as water, from their host plants, thus inflicting physiological stresses to the latter, leading to stunting, loss of productivity, and sometimes death, of the host crops [1]; [2]. While some parasitic plants are facultative parasites capable of independent life styles in the absence of host plants, others like dodder (*Cuscuta* spp.) are obligate parasites that rely entirely on host plants for completion of their life cycles [1]; [7]. Parasitic plants typically extract nutrients from their host plants through specialized organs referred to as haustoria [3]; [4]. While the anatomical details of haustoria have been extensively studied, the molecular signaling events that condition the establishment of haustoria are not well understood. Recent studies suggest that host plants respond to initial invasion by parasitic plants with defense responses that share similar features with anti-microbial defenses [5]; [6]. However, it remains to be resolved how parasitic plants overcome these defense responses to establish successful parasitism. Understanding the molecular basis of parasitic plant-host interactions is of critical importance to crop production as many staple crops, including maize, tomato, alfalfa, and soybean, are known to suffer from infestation by parasitic plants, leading to significant yield losses [1]; [2].

Parasitic plants in the genus *Cuscuta*, family *Convolvulaceae*, commonly referred to as dodders, are ideal models for studying plant-parasite interactions [7]; [8]. Over 100 species of dodders have been found to be distributed worldwide, causing damages to trees and shrubs in natural habitats as well as many cultivated crops [1]; [7]. The dodder-host relationship is easy to observe as dodders, unlike root parasites, establish readily visible hausteria on the aerial parts of their hosts [3]; [4]; [11]. The obligate nature of dodder parasitism also permits focused examination of the parasitic relationship without the interference of other forms of nutrition requisition. In addition, dodders are well suited for biochemical as well as cell biological analysis as, once established on a host, it is relatively easy to acquire large quantities of dodder tissue, and the physical structure for parasitism (haustorium) is abundantly distributed along the parasite-host interface [3]; [4]; [11]. The relatively broad host range of most dodder species further permits the identification of shared characteristics through comparative analysis [7]; [9]; [10]. Finally, dodders are economically important pests as they cause damages to many crops including tomatoes and alfalfa, as well as diverse species in natural habitats. Therefore, it is expected that knowledge obtained through the examination of dodder-host plant interactions will provide the basis for an improved understanding of how parasitic plants in general invade their hosts.

Gaining molecular insights of the genetic make-up of dodders constitutes one of the key steps toward the goal of understanding the mechanism of dodder-host interactions. In addition to the potential basic research implications, knowledge of molecular genetics of dodder is expected to have practical applications by providing components/pathways for targeted control measures. This is particularly relevant in light of the recent discovery of RNA silencing (or RNA interference, RNAi), an RNA-based surveillance mechanism highly conserved in eukaryotic organisms [12]; [13]. The RNAi machinery uses double-stranded RNA (dsRNA) as the template to produce small RNAs (small interfering RNAs or siRNAs) that in turn target other homologous RNAs for degradation or translational repression in a sequence-specific manner, down-regulating the expression of the corresponding genes [14].

Importantly, RNAi in plants produces a systemic silencing signal, which travels through the vascular bundles to silence homologous RNA in the whole plant, even if the silencing-inducing dsRNA is introduced to just a few leaves [15]; [16]. Since dodder is known to acquire the contents of the host vascular bundles with high efficiencies, the systemic silencing signal generated in the host plant would be expected to be acquired by dodder as well [17]; [18], with the potential of exerting the silencing effect in dodder. Indeed, Tomilov and colleagues [19] showed that siRNAs generated from a dsRNA-expressing transgene readily moved from the lettuce host to the parasitic plant *Triphysaria versicolor* and caused the silencing of a homologous transgene. More recently, host-induced RNAi has been shown to effectively suppress the dodder parasitism by targeting haustorium-residing gene transcripts [20]. However, success of this RNAi-based management strategy relies on the availability of sequence information of dodder genes, especially sequences of genes that are important for parasitism.

We have initiated the current study of profiling mRNAs of two different dodder species with two goals in mind: (i) laying the groundwork for future in-depth investigation of the interaction mechanisms between dodders and their host plants, and (ii) identifying targets for RNAi-based control of dodder parasite. To this end, we have used the 454 high throughput sequencing platform to analyze the mRNAs of *Cuscuta pentagona* and *C. suaveolens*, two common dodder species in America. Our efforts resulted in the assembly of over 46,000 isotigs and contigs for both of the dodder species. Bioinformatic analysis of these assembled sequences, hereafter collectively referred to as expressed sequence tags (ESTs), predicted that more than half of them encode proteins homologous to known plant proteins. In addition, comparing the assembled ESTs with transcripts of 12 other plants with annotated genomes suggests that among these better known plants, dodder is most closely related to tomato. Finally, by applying a rigorous set of filtering criteria, we were able to identify a small set of dodder ESTs that are likely related to the parasitic life style. In conclusion, this study offered the first glimpse of the genome characteristics of dodders, laying the foundation for further in-depth examinations in the future.

## Results and Discussion

### Profiling transcriptomes of two dodder species with high throughput sequencing - *de novo* transcript assembly and quality assessment

To begin to understand the genetic makeup of the parasitic plant dodder, we chose to analyze the expressed genes of two different *Cuscuta* species – *C. pentagona* and *C. suaveolens. C. pentagona* counts tomato as its primary host, whereas *C. suaveolens* frequently parasitizes alfalfa [21]; [22]. The seeds of *C. pentagona* and *C. suaveolens* were kindly provided to us by Drs. Tom Lanini at University of California-Davis, and Peter Reisen at Forage Genetics International in Indiana, respectively. We isolated polyadenylated RNAs from both of the dodder species colonized on alfalfa plants, and subjected the isolated RNAs to high throughput sequencing with the Roche 454 platform (Purdue University, IN).

**Figure 1:**
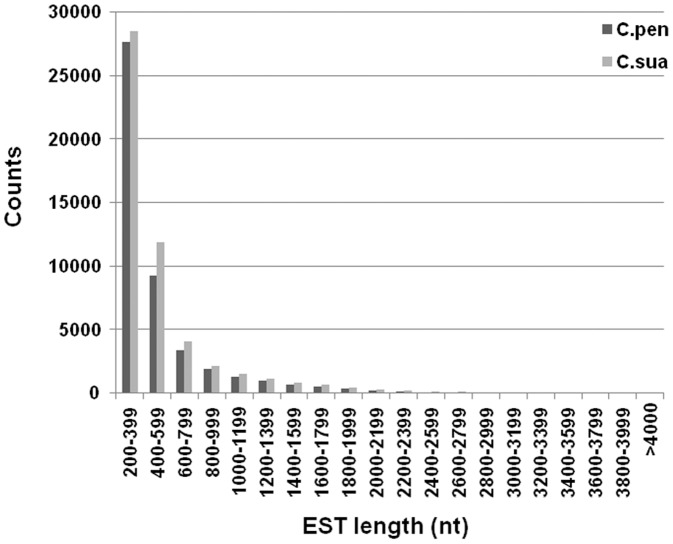
Size distribution of assembled *C. pentagona (C. pen)* and *C. suaveolens (C. sua)* ESTs. The X-axis depicts the sizes of ESTs divided in 200 nt increments. ESTs smaller than 200 nt were excluded, and those large then 4,000 nt were combined. The Y-axis represents the number of ESTs falling into each size categories.

A total of 762,066 and 786,103 sequence reads were obtained for *C. pentagona* and *C. suaveolens*, respectively, with an average read size of 271 bases (Table 1). Initial *de novo* assembly of raw reads using the Newbler 2.5 software (see Materials and methods section for details) showed that a substantial proportion of reads of both species could be assembled into large isotigs of 1,000 nucleotides (nt) or longer, resulting in 18,150 and 20,510 isotigs for *C. pentagona* and *C. suaveolens*, respectively. Isotigs that share mostly identical sequences interspersed with short range differences were presumed to represent splice variants of the same genes, thus were further sorted into isogroups. This reduces the total numbers of isotigs to 14,411 for *C. pentagona* and 15,511 for *C. suaveolens* (Table 1). The remaining reads not assembled into isotigs were subjected to a secondary assembly attempt with the CAP3 software [23], which yielded large numbers of shorter contigs and singletons. To eliminate sequences with low coding probabilities and to speed up bioinformatic operations, we excluded those that are shorter than 200 nucleotides (nt) in length from further analysis. These additional assembly and filtering steps resulted in 15,292 and 15,232 additional CAP3 contigs, and 16,867 and 21,439 unassembled singletons, for *C. pentagona* and *C. suaveolens*, respectively (Table 1). Altogether, these two rounds of *de novo* assembly produced 46,570 and 52,182 unique ESTs for *C. pentagona* and *C. suaveolens*, respectively. The size distribution of these ESTs is depicted in Fig. 1. The raw sequence data of *C. pentagona* and *C. suaveolens* have been deposited in GenBank with the accession numbers SRA071492 and SRA071493; and their assembled ESTs were deposited in GenBank's TSA database with the accession numbers SUB373257 and SUB357373.

**Table 1:** *C. pentagona* and *C. suaveolens* transcriptome statistics.

EST types	*C. pentagona*	*C. suaveolens*
Total raw reads	762,066	786,103
Total bases	196,053,201	224,331,811
Isotigs prior to isogrouping	18,150	20,510
Isotigs after isogrouping	14,411	15,511
Average isotig size	921	1,042
N50 isotig size	1,112	1,276
Largest isotig	9,410	10,709
CAP3 contigs	15,292	15,232
Singletons	16,867	21,439
Total unique ESTs	46,570	52,182

Next, to assess sequence similarities between the putative proteins encoded by dodder ESTs and known proteins of other plants, we subjected the EST sequences to similarity searches against proteins encoded by genomes of 12 different plant species using the BLASTx algorithm, with a confidence threshold of 1e-6. These 12 species (Table 2, left column) were chosen for this comparison because their genomes have been sequenced, providing more comprehensive coverage of their protein-coding potentials. Conversely, plant species with partially sequenced genomes, often in the form of transcriptomes, were excluded to avoid potential biases. These excluded species range from other parasitic plants ([46]; Parasitic Plant Genome Project website (http://ppgp.huck.psu.edu/), to sweet potato which is a close relative of *Cuscuta* species [24]; [35]. However, they were included in later analyses aimed at identifying transcripts shared by parasitic plants (see later).

**Table 2:** Comparison of putative dodder proteins with proteins of other plants.

Plant species included	Sequence source	# of predicted dodder proteins most similar to proteins of the respective plant	
		*C. pentagona*	*C. suaveolens*
Tomato	SGN	13,554	13,938
Grapevine	JGI	4,099	4,235
*Mimulus guttatus*	JGI	3,772	3,730
Poplar	NCBI RefSeq	2,535	2,478
Soybean	NCBI RefSeq	604	692
*Arabidopsis*	TAIR	592	647
Rice	NCBI RefSeq	435	511
*Brachypodium distachyon*	JGI	183	207
Maize	NCBI RefSeq	159	202
*Selaginella moellendorffii*	JGI	43	46
*Physcomitrella patens*	JGI	38	58
*Chlamydomonas reinhardtii*	JGI	13	22
Total		26,027	26,766

The numbers in Table 2 represent the counts of putative dodder proteins that show highest similarities with proteins of the respective plant species. For example, 13,554 putative *C. pentagona* proteins are more similar to their tomato orthologs than to those in other 11 species. Conversely, 4,099 putative *C. pentagona* proteins are more similar to their grapevine orthologs than to those in other 11 species including tomato. Together about 56% (26,027/46,570) of *C. pentagona* and 51% (26,766/52,182) of *C. suaveolens* ESTs have at least one hit to known plant proteins included in our comparison [25]. These numbers are considerably higher than other recently profiled species [26], suggesting a satisfactory depth of the sequencing efforts.

We also used another commonly used approach to further confirm the quality of the sequence data [27]–[29]. This alternative approach compared the assembled dodder ESTs with *Arabidopsis* transcripts derived from single copy genes that are highly conserved across all eukaryotes, designated ultra-conserved orthologs (UCOs). Out of 357 *Arabidopsis* UCOs, 350 (∼98%) and 355 (∼99%) had hits in *C. pentagona* and *C. suaveolens* EST datasets, respectively, suggesting that our sequencing effort has captured most of expressed sequences.

**Figure 2:**
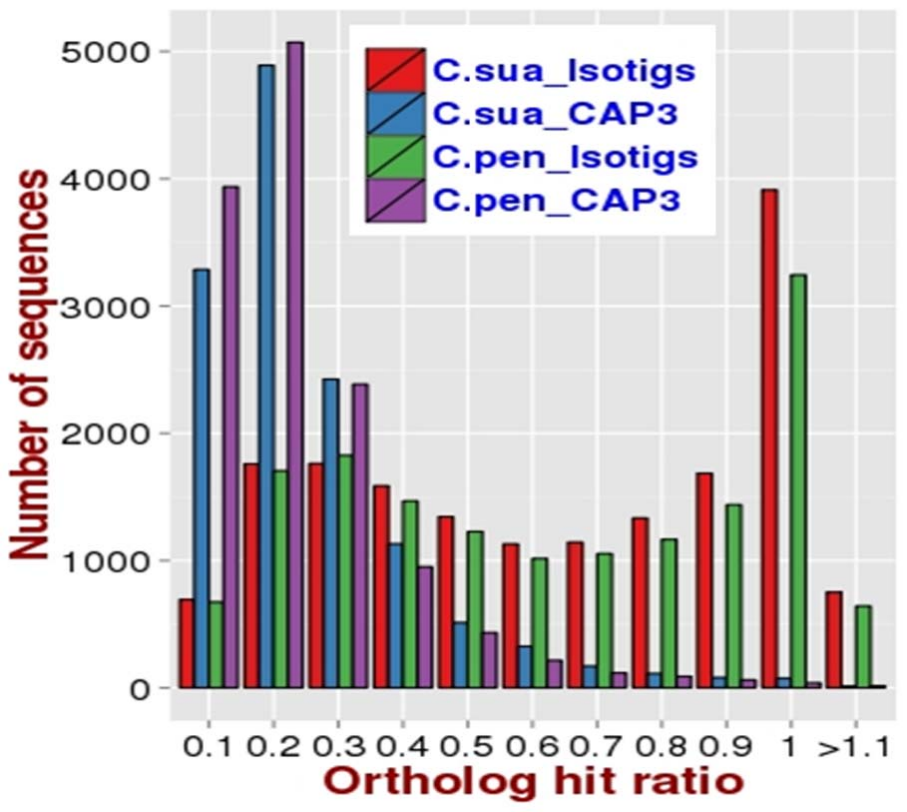
The lengths of *C. pentagona (C. pen)* and *C. suaveolens (C. sua)* ESTs as estimated by the calculating ortholog hit ratio (OHR; see text for detailed description). ESTs with OHR approaching 1 are considered likely to be of full lengths.

Finally, we also determined to what extent the assembled ESTs represent full length mRNAs, using a method described by O'Neil and colleagues [30]. This procedure uses the assembled ESTs as queries to identify their full length orthologs in a closely related, fully sequenced species, and subsequently calculates the ortholog hit ratio (OHR) for each EST by dividing the length (in number of bases) of the continuously aligned EST with the length of the corresponding ortholog. An OHR value close to one indicates a fully assembled sequence, whereas a value close to zero indicates a partially assembled transcript. We calculated OHRs for dodder ESTs with tomato cDNAs as references and found that a majority of the isotigs had OHRs indicative of near complete sequences. As depicted in Fig. 2, out of 16,107 *C. pentagona* isotigs that have tomato orthologs, 10,350 (∼64%) had an OHR of 0.5 or higher. Similarly, out of 17,859 *C. suaveolens* isotigs that have tomato orthologs, 11,959 (∼67%) showed an OHR of 0.5 or higher. Conversely, among CAP3 assembled sequences and singletons, only 7% (984/13,335) of *C. pentagona* and 10% (1300/13039) of *C. suaveolens* sequences showed an OHR of more than 0.5, indicating that most of these shorter ESTs are partial sequences.

### Predicted dodder proteins show high levels of similarity to tomato proteins

**Figure 3:**
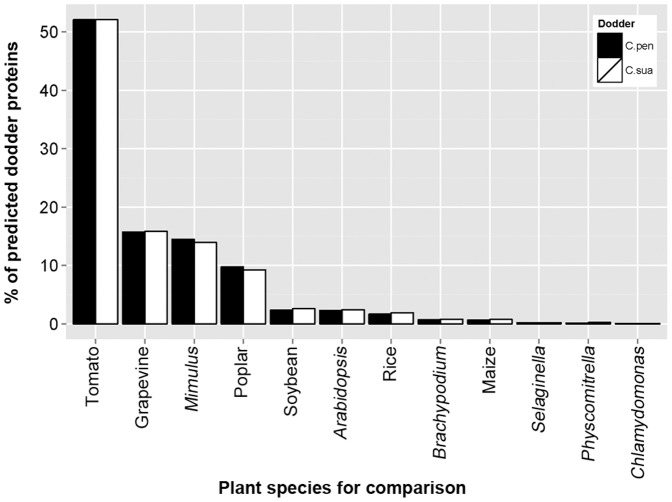
Proportion of dodder ESTs sharing the highest similarities with mRNAs of each of the twelve non-parasitic plant species.

As described in the previous section, comparison of assembled dodder ESTs with the transcriptomes of 12 plant species with sequenced genomes revealed a high level of similarity between dodder and tomato (Table 2). Indeed, among the 26,027 *C. pentagona* and 26,766 *C. suaveolens* ESTs predicted to encode proteins that are highly similar to proteins of the 12 plants, about 52% of them (13,554 and 13,938 for *C. pentagona* and *C. suaveolens*, respectively; Table 2) displayed the highest similarity to tomato proteins (Fig. 3). By contrast, only about 16% of predicted dodder proteins displayed the highest similarity to proteins of grapevine, the next closest species (Table 2 and Fig. 3). This analysis suggests that among the plant species with sequenced genomes, dodder is most closely related to tomato. These results are consistent with the fact that dodder and tomato belong to two phylogenetically related plant families (*Convolvulaceae* and *Solanaceae*) of the same order (*Solanales*) [31]; [32]. Interestingly, about 2% of the dodder ESTs showed higher similarities to transcripts of monocots, early land plants and algae (data not shown), suggesting that some of the dodder sequences might have been acquired from more distantly related species through horizontal gene transfer events [33]; [34].

### Annotation of assembled sequences

**Figure 4:**
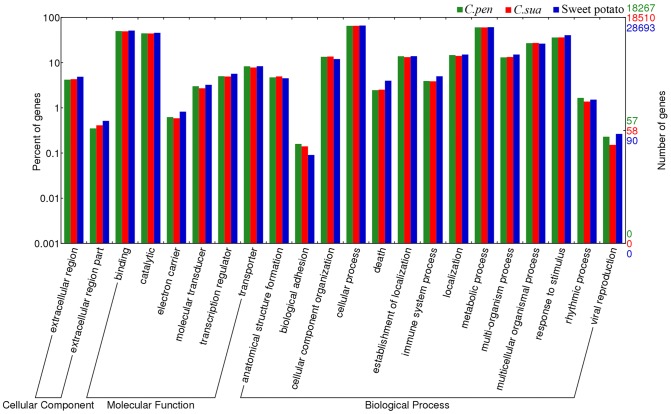
Gene Ontology categories of assembled dodder ESTs based on biological functions, with the transcriptome of sweet potato as reference. The various GO categories are listed along the X-axis, whereas the Y-axes (both sides) represent two different ways to showcase the number of ESTs in each GO category. On the left side, the numbers were converted to percentages of the total annotated ESTs, whereas on the right side actual numbers were plotted. Note that the scales on both Y-axes are logarithmic.

Having confirmed the quality of the EST assemblies, we then tried to annotate the *C. pentagona* and *C. suaveolens* transcriptomes. For this purpose, we used as reference the newly available transcriptome data of sweet potato as it belongs to the same plant family (*Convolvulaceae*) as dodder, is hence among the closest free-growing relatives of dodder [24]; [35]. Additionally, similar to dodder transcriptiome assemblies, that of sweet potato was also derived from high throughput sequencing efforts. Consequently, both datasets could be analyzed in parallel to permit more meaningful comparison. We subjected the dodder as well as sweet potato ESTs to BLASTx searches against proteins sequences of other better known plant species as described in the Materials and Methods section. The Gene Ontology (GO) categories were then assigned using Blast2GO, and visualized using the WEGO annotation plotting tool [36]. A total of 18,627 *C. pentagona* and 18,510 *C. suaveolens* ESTs were annotated and their GO terms were found to be distributed in a variety of categories and didn't show significant differences between the two species (Fig. 4). Similar analysis permitted the annotation of 28,693 sweet potato ESTs. We then compared the GO terms for these three species using the WEGO tool kit (Fig. 4). As shown in Fig. 4, overall transcripts of these three species share very similar distribution patterns in terms of genes involved in various functions. The only notable differences are among genes that are involved in three categories of biological processes. Notably, genes involved in biological adhesion are slightly overrepresented in the two dodder species, which could be related with their parasitic life style. Conversely, genes involved in cell death and immune system process are slightly underrepresented in the dodder species, for unknown reasons (Fig. 4). The overall similarity of GO terms between the parasitic dodder plants and the free living sweet potato suggest that relative few differences exist that could account for the distinct life styles of the two types of plants (also see later). This is not surprising as dodder and sweet potato belong to the same family of flowering plants (*Convolvulaceae*). Other much more divergent plants were found to contain relatively few differences at the genome level as well [37]. It is hence thought that plants likely adapt to new life styles through acquisition of new functions by existing genes [37]; [38].

### Transcripts of the host plants were absent in dodder transcriptomes

Previous reports suggested that mRNAs of host plants could translocate to dodder tissues [39]. We hence evaluated the extent of possible mRNA migration from the host plant (alfalfa, *Medicago sativa*) to dodder by comparing the assembled dodder ESTs with the ca. 15,000 alfalfa mRNA sequences available at GenBank. We failed to find any dodder EST that matches alfalfa mRNAs with satisfactory confidence, suggesting that the migration of mRNA from host to dodder probably occurred at very low frequencies, thus could have escaped the detection by our sequencing approach (data not shown). However, since our mRNA samples were derived from distal tendril sections unattached to hosts (see Materials and Methods for details), our results do not rule out the possibility that host mRNA might be present in dodder tissues in or around haustoria [39].

### Dodder genes potentially involved in anti-microbial defense

Since dodders are promiscuous parasitic plants that frequently have wide host ranges, they are likely exposed to a large number of plant microbial pathogens. Examples of reported microbial pathogens of host plants that are present in dodders have included viruses, bacteria, and phytoplasma [40]–[43]. Hence, dodders are thought to have relatively robust anti-microbial defense capabilities. To evaluate this possibility, we have searched the dodder ESTs for genes involved in RNA silencing pathways known to play important roles in antiviral defense, with the four dicer-like dsRNA nucleases (DCLs) of *Arabidopsis* as queries. We found 31 *C. pentagona* and 39 *C. suaveolens* ESTs that share significant sequence similarities to *Arabidopsis* DCLs. However, these contigs are relatively short (about 500 bases) compared with the expected size of typical DCL transcripts (ca. 4 kb), preventing us from classifying them into distinct DCL families with high confidence (data not shown). This is not surprising as DCL mRNAs are known to accumulate at relatively low levels [44]. Nevertheless, the presence of a large number of dodder ESTs in both species with DCL signatures suggests that the respective DCLs are transcribed and likely represent functional genes.

### Sequences of virus origins

Dodder has long been known to transmit viruses and other microbial pathogens between its host plants [40]. It was speculated that dodders could support symptomless infections of some of the viruses, thus serving as reservoirs for these viruses [41]. To determine whether sequences with characteristics of plant viruses are identifiable in our dodder EST libraries, we searched the datasets for virus-specific sequences. Interestingly, multiple contigs in both libraries were found to be of virus origins, which can be broadly grouped into two categories. The first category consists of 34 contigs (18 from *C. pentagona*, 16 from *C. suaveolens*) that are highly homologous to *Impatiens necrotic spot virus* (INSV), an ambisense plant virus frequently associated with horticultural and greenhouse plants. Unfortunately, we did not examine whether INSV was also present in the alfalfa plants used as host for dodder propagation. Nevertheless, the identification of INSV reads in both dodder species clearly indicates that INSV is abundantly present in dodder tissues, and suggests that dodders could be efficient vehicles for INSV transmission.

The second category of virus sequences was comprised of four sequences (three in *C. pentagona* and one in *C. suaveolens*) that share significant homology with various plant cryptic viruses. Cryptic viruses are dsRNA viruses that rarely cause any disease symptoms in host plants [45]. They are unique in that they are incapable of cell-to-cell movement in the host plants, thus relying entirely on cell division for their intra-plant dissemination. Plant cryptic viruses have been found mostly in wild plants [45]. All four sequences identified in the dodder libraries share amino acid level similarities with the capsid protein genes of a number of known plant cryptic viruses, including *Raphanus sativus cryptic virus 1* (RSCV1), *Beet cryptic virus 1* (BCV1), and *Rose cryptic virus 1*(RCV1; see Table 3). Furthermore, sequence similarity with known cryptic viruses was even detected at the nucleotide levels for one of the ESTs (Table 3; sequences available in Section I of Text S1). These results suggest that the cryptic virus(es) in dodders are related to known cryptic viruses. Further investigations are needed to determine the nature of cryptic viruses present in dodders.

**Table 3:** Cryptic virus signatures in dodder sequence libraries.

Sequence ID	Length (nt)	Origin	Hits (CP of)	Accession number	Nucleotide Identity	Amino acid Identity	Amino acid similarity
GWFM1H202FY4RF	455	*C. pentagona*	RSCV 1	ABA46820	Undetectable	43% (48/111)	69% (77/111)
GWFM1H202INAQY	223	*C. pentagona*	BCV 1	YP_002308575	76% (82/108)	61% (34/56)	82% (46/56)
Contig2210	402	*C. pentogona*	RCV 1	YP_001686788	Undetectable	44% (42/96)	55% (53/96)
Contig2599	473	*C. suaveolens*	RSCV 1	ABA46820	Undetectable	49% (68/140)	73% (102/140)

### Potential dodder genes associated with parasitism

One of the goals of this study was to assess whether the parasitic life style of dodder is associated with unique genes and whether these genes can be identified through analysis of dodder transcriptomes. To resolve these questions, we hypothesized that genes required for the parasitic life style would be conserved among various parasitic plant species. Thus, we undertook a three step bioinformatic screening to identify potential dodder ESTs that encode proteins conserved among parasitic plants. First, we identified 25,411 EST pairs from *C. pentagona* and *C.suaveolens* datasets that are highly homologous and thus considered to be orthologous. This set of dodder ESTs were then subjected to tBLASTx searches against the transcriptomes of 12 non-parasitic plants (Table 2) to eliminate those homologous to protein-coding sequences of these plants. A new subset of ESTs was identified that did not appear to share significant sequence similarities with these non-parasitic species, hence were considered to be unique to the two dodder species.

We then assessed whether this subset of dodder ESTs contained transcripts that are shared by other parasitic plants by using this set of dodder ESTs as queries to search the assembled transcriptomes of three other parasitic plants, *Triphysaria versicolor*, *Striga hermonthica*, and *Orobanche aegyptiaca* ([46]; Parasitic Plant Genome Project (http://ppgp.huck.psu.edu/). As a result, 96 pairs of dodder ESTs were found to share significant (E = −6) similarity with transcripts of all three parasitic plant species. These EST pairs were then subjected to another round of tBLASTx search against the NCBI nonredundant nucleic acids database to eliminate any transcripts that share similarities with genes of other free-living plants, but were missed in the first round of screening. This left us with 46 pairs of dodder (*C. pentagona* and *C. suaveolens*) ESTs that didn't show significant similarities to other non-parasitic plants. We then further narrowed down the number of candidate ESTs using two additional criteria. First, we excluded EST pairs with either one or both sequences shorter than 400 bases. Second, we manually inspected the remaining ESTs for their potential translatability and removed the ESTs that contained multiple stop codons in all six possible frames. These filtering steps led to the identification of seven pairs of ESTs (14 sequences in total) with sizes ranging from 414 to 2579 nts (Table 4; see Table S1 for an expanded version of Table 4 containing sequences).

**Table 4:** Candidate ESTs with possible roles in the parasitic life style.

C. pentagona	size	C. suaveolens	size	Orobanche	Triphysaria	Striga
000531_C-pen_isotig01704	2110	000530_C-sua_isotig00937	2200	OrAeBC4_64469	TrVeBC2_265167	StHeBC2_11136
000531_C-pen_isotig06345	1589	000530_C-sua_isotig08543	2579	OrAeBC4_26152	TrVeBC2_61808	StHeBC2_33600
000531_C-pen_isotig12615	651	000530_C-sua_isotig16127	606	OrAeBC4_65586	TrVeBC2_54883	StHeBC2_11136
000531_C-pen_isotig16431	417	000530_C-sua_isotig04561	1632	OrAeBC4_508124	TrVeBC2_268016	StHeBC2_29409
000531_C-pen_isotig16457	414	000530_C-sua_isotig17995	504	OrAeBC4_608131	TrVeBC2_260759	StHeBC2_293425
Singleton cluster_2020	489	000530_C-sua_isotig13395	839	OrAeBC4_1059522	TrVeBC2_266559	StHeBC2_33600
Singleton cluster_3966	433	Singleton cluster_1638	553	OrAeBC4_65586	TrVeBC2_265167	StHeBC2_11136

**Figure 5:**
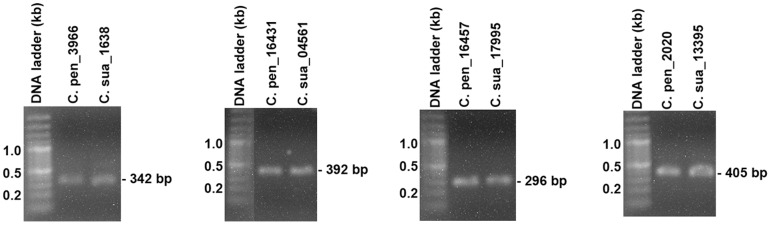
Confirmation of four pairs of dodder ESTs with RT-PCR. Four pairs of *C. pentagona (C. pen)* and *C. suaveolens (C. sua)* ESTs were chosen for assessment, generating fragments of 342, 392, 296, and 405 bps, respectively. Sequences of the primers used are provided in Section II of Text S1.

We next used reverse-transcription PCR (RT-PCR) to confirm the presence of these transcripts in the polyadenylated RNAs of both *C. pentagona* and *C. suaveolens*. As shown in Fig. 5, all four pairs we chose to evaluate yielded PCR fragments of expected sizes with both *C. pentagona* and *C. suaveolens* polyadenylated RNA as templates. These results strongly suggest that mRNAs corresponding to these ESTs are transcribed in both dodder species. Accordingly, the genes that encode these mRNAs must be part of dodder genomes. These genes could be tested in future experiments for their potential to serve as tragets of RNAi-based engineering of dodder resistance.

### Concluding remarks

In this report, we documented an analysis of the transcriptomes of two obligatory parasitic plant species, *C. pentagona* and *C. suaveolens*. Our data suggest that while an overwhelming majority of genes of these parasitic plants are homologous to other free-living plants, a small set of genes could be identified that appear to be shared by parasitic plants only. More careful examination is needed to confirm their relationship with the parasitic life style of dodder. Once confirmed, these genes could become potential targets for future management attempts aimed at controlling these pests of crop plants.

## Methods

### Plant growth, library preparation, and sequencing

*C. pentagona* seed was provided by Dr. T. Lanini at the University of California-Davis, whereas *C. suaveolens* seed was a gift from Dr. P. Reisen at Forage Genetics International, Indiana. These seeds are available to interested researchers. Both *C. pentagona* and *C. suaveolens* materials were propagated in greenhouse at OARDC, OSU, with alfalfa as the host. To avoid contamination of host plant tissues, only distal portions of dodder tendrils that were at least 10 cm away from host plants, including growing meristems and flowering buds, were collected and pooled together for total RNA extraction using the TRIzol Reagent (Invitrogen, Carlsbad, CA) following the protocol provided by the supplier. Total RNA samples were shipped to the Purdue University Genomics Core Facility (PUGCF) on dry ice for mRNA sequencing using a Roche GS-FLX (454) platform. Isolation of mRNA, fragmentation, library preparation, sequencing and assembling were performed by PUGCF staff.

### Sequence preprocessing, transcriptome assembly, and coverage assessment

Raw sequences were assembled at PUGCF using Newbler 2.5 program. The secondary assembly of the remaining singletons was carried out using the CAP3 software [23] with the ‘z’ value set to one. To assess the quality of assembly for ESTs larger than 200 nt, we retrieved the sequences of Arabidopsis single copy proteins deemed highly conserved across all eukaryotes from the following website: http://compgenomics.ucdavis.edu/compositae_reference.php, and compared these sequences with assembled *C. pentagona* and *C. suaveolens* ESTs using the tBLASTn software package, with the e-value cut-off set at 1e-6, and the “max_target_seqs” option set to 1 [47]. Additionally, the assembled dodder ESTs were compared with tomato protein sequences [ITAG2.3 released by International Tomato Genome Project in August 2011, made available through Solanum Genome Network (SGN; http://solgenomics.net/)] using the BLASTx software (e-value cut-off = 1e-6). A custom python script first described by Ewen-Campen and colleagues [48] was used to calculate the ortholog hit ratio (OHR) between dodder sequences and tomato proteins.

### Functional annotation and classification

To cut down the time needed for the large scale BLAST searches, we included only the longest isotig from each isogroup of a given dodder species for further comparisons [47]. In addition, we used the recently assembled sweet potato ESTs as reference to gauge the accuracy of our annotations [35]. The dodder ESTs were searched against protein sequences of *Arabidopsis,* grapevine (*Vitis vinifera*), tomato, rice, and other reference sequences in the RefSeq database using the BLASTx program with the e-value cut-off set at 1e-6. We then used the Blast2GO (v.2.5.0) program [49] to map Gene Ontology (GO; [50]) terms and annotate the dodder sequences using blast results (e-value filter of 1e-6, annotation cutoff of 45, and GO weight equal to 5). The GO terms were exported to WEGO GO plotting tool [17] and categorized using level 2 of the GO lineage.

### Sequence comparisons

To elucidate the evolutionary relationship between dodders and other fully sequenced plant species, we subjected the dodder ESTs to a BLASTx search against the predicted proteomes of nine sequenced plants: poplar (*Populus trichocarpa*), *Medicago truncatula*, *Arabidopsis*, maize (*Zea mays*), rice, tomato, *Selaginella*, *Physcomitrella patens*, *Chlamydomonas reinhardtii*, and *Mimulus guttatus*. To find sequences common to both dodder species, we did reciprocal blast searches using the BLAST+ software with 1e-15 as the e-value cutoff [24]. The BLAST results were combined using a custom python script and unique putative ortholog pairs were retrieved for further analysis. We then compared the dodder-specific ESTs with those of three other parasitic plants, namely *Triphysaria versicolor*, *Striga hermonthica*, and *Orobanche aegyptiaca*, whose EST data are available through the Parasitic Plant Genome Project website (http://ppgp.huck.psu.edu/). The software used was tBLASTx [47], with1e-6 as the E-value cutoff.

## Supporting Information

Table S1 ESTs shared by parasitic plants – with sequences.
(XLSX)

Text S1 ESTs of putative cryptic viruses, and sequences of oligos used in this study. **I**: EST sequences matching cryptic viruses. **II**: Sequences of oligos used to confirm the expression of some dodder ESTs.
(DOCX)
