# Supplementary material for: Profiling mRNAs of Two Cuscuta Species Reveals Possible Candidate Transcripts Shared by Parasitic Plants
Source: PLoS One. 2013 Nov 27;8(11):e81389. doi: 10.1371/journal.pone.0081389 (PMC3842250; doi:10.1371/journal.pone.0081389)
Supplement: Text S1 — ESTs of putative cryptic viruses, and sequences of oligos used in this study. I: EST sequences matching cryptic viruses. II: Sequences of oligos used to confirm the expression of some dodder ESTs. (DOCX) [file pone.0081389.s002.docx]

**I: EST sequences matching cryptic viruses**

>000531_C-pen_GWFM1H202FY4RF

Agatttggaaatgttactccttcaatctcccaacgccctggctggacccgcgcttcccactacacctttgggcacatgaccactcatttgcccaatttgagcttctacttttctcgtctgtactctgtttgtgatgctgctacccgcgacggaactaccgaaatctcttttgctagagatgttgatggtcctcgcttcttggccactatatttggtcgagcatgtgacaatgaagctactgctgagcacatcatgctccgctcccccggtgcctctcttgtttatgctggtaaccttcatctctgccaaaaagctgcccttcaaatgaacagtgaactccctggcaatcttaacattgacaatggtaaatgtagagaattcttgggtctccgctctccgcctcgaccattcattttactgtctcacggttgctgctatgatggcaagatattgcc

>000531_C-pen_GWFM1H202INAQY

cagggagccatggaggacagccctgcgtgaagcgagcggtagagaccatcaattgatcgcagaggccaagcacgtggaacatcatttggctgtcagggacataggtgtttacgtcacgactggggacagtgtgtaacggataagcataagcaatctccagtaaaggaggaacatgatcagcatcaatgagcccagtggcagcaaagcggctgcgttgattagg

>Contig2210 (C-pen)

atcgtcgcgggtcaattgtattaactatggctgcgctacttgttcccaaaagcttacctaatctacatcgtccagccctaattcttgctcccctttgttgaagctattcagaactttggtcctttcaacccaaccagtattcctcagaactacctctgcgtgcctgtttatcctgaaaacacgcttaatgaaggcagaaccgccgctcagtggagctccttggaatatgaagccaccattccaggaatgaaagagattgggatccctttcaagtctgtggacaccggtgtcaaatccgggactgcttggtggtgtatgcactatgaaagcatggcaacagccatgacattgtgtgcctgtacccgcccattaactactctgatcactcggtccttcttcaag

>Contig2599 (C-sua)

Ctttatcctctgtctaccctctggatccctggccctctagtcagtttcttcaagaacctggcttgcttcaccccggaccctgaaagatttggaaatgttactccttcgatctgccaacgccctggctggacccgcgcttcccactacacctttgggcacatgaccactcatttgcccaatttgagcttctacttttctcgtctctactctgtttgtgatgctgctacccgcgacgggacaaccgaaaccccttttgctagagatgttgatggtcctcgcttcttggccactatatttggtcgagcatgtgacaatgaagctactgctgaacacatcatgctccgctcccccggtgcctctcttgtttatgctggtaaccttcgtctctggcaaaaagctgcccttcaaatgaacagtgaactccctggcaatcttaacattgacaatgatgatgtagaggattctgggtctcg

**II: Sequences of oligos used to confirm the expression of some dodder ESTs:**

For C. pen_3966 and C. sua_1638: 5’-CCCCACCACGCTCATATT-3’ and 5’-CAAGTTCATTCCTCGCCTTG-3’;

For C. pen_16431 and C. sua_04561: 5’-TCAACTTCTTCTTCCATTCCATC-3’ and 5’-CGAGGCAAGTCGTCTCAGAT-3’;

For C. pen_16457 and C. sua_17995: 5’-TGTGCACCTCAGCATCTTCT-3’ and 5’-TTGAAAGGATTGGGTTTGGA-3’;

For C. pen_2020 and C. sua_13395: 5’-TGCAAGCTTGTTGTGGATCT-3’ and 5’-CAGGTCCACGTCAATGAAAA-3’
